# A survey-based study about burnout among postgraduate medical trainees: implications for leaders in healthcare management

**DOI:** 10.3389/fpubh.2023.1209191

**Published:** 2023-07-12

**Authors:** Aline Yacoubian, Evangelia Demerouti, Jad A. Degheili, Albert El Hajj

**Affiliations:** ^1^Department of Surgery, American University of Beirut Medical Center, Beirut, Lebanon; ^2^Dept. Industrial Engineering & Innovation Sciences, Eindhoven University of Technology, Eindhoven, Netherlands; ^3^Department of Urology, Children's Hospital of Eastern Ontario (CHEO), Ottawa, ON, Canada

**Keywords:** burnout, postgraduate medical trainees, wellbeing, wellness, leadership

## Abstract

**Introduction:**

The goal of the study is to assess burnout among postgraduate medical trainees, evaluate the association with sociodemographic features and offer potential wellness strategies for leaders responsible for their education, training, management, and wellbeing.

**Methods:**

The Oldenburg Burnout Inventory was used. The web-based, voluntary, and anonymous survey was sent to postgraduate medical trainees from various specialties and all years of training in a tertiary medical center in Beirut, Lebanon. Additional questions were added after the survey regarding reporting channels for burnout and possible interventions for wellbeing.

**Results:**

The total number of valid responses are 188. The prevalence rates of high burnout are 37.2% for disengagement and 51.1% for exhaustion. There is a significant difference between the mean of exhaustion and gender (*p* = 0.003). There is a significant difference between the mean of disengagement and year of training (*p* = 0.017). There is a significant difference between the mean of exhaustion and year of training (*p* = 0.029). There is a significant difference between the frequency of disengagement and year of training (*p* = 0.027).

**Conclusion:**

The study reveals how postgraduate medical training program is impacted by the existing challenges from social, health, and financial standpoint, along with the instabilities encountered such as multiple wars and port blast in 2020 and how these variables aggravate burnout. Burnout severely impacts the education and training of PGMT and promoting wellbeing can help reverse the process. Findings contribute to establishing effective strategic interventions for leaders in healthcare management to adopt.

## Introduction

Burnout is a psychological syndrome and an occupational problem provoked by long-lasting interpersonal stressors (1). The keyword emerged in a novel called “A Burnt-Out Case” by Graham Greene with its English version published in 1961 (2) but was not related to occupation back then. Herbert Freudenberger used the term as a popular metaphor and concept for mental exhaustion experienced by the social volunteers who worked in the Free Clinic movement that he created in San Francisco, the United States (US) in 1967 to serve the young population (3). Freudenberger mentioned that he used the definition of burnout in the article from “the dictionary” without specifying the name of the dictionary in the references (2, 3). He defined burnout as failing, wearing out, or becoming exhausted due to applying a lot of energy, strength, and resources (3). Burnout is a condition of depersonalization, emotional exhaustion and low personal accomplishment and can cause deteriorated quality of care or service, personal dysfunction, fatigue, insomnia and other problems (4).

The medical field has experienced a rapid upsurge in mental ailment and burnout due to the coronavirus disease 2019 (COVID-19) pandemic with a negative impact on the residency and fellowship training (5). Burnout is common in medicine with an estimated $4.6 billion a year linked to physician turnover and decreased productivity caused by physician burnout in the US (6). Costs pertinent to burnout are greater in the younger section of physicians in the US (those aged under 55 years) (7). Burnout was added in the 11th Revision of the International Classification of Diseases (ICD-11) as a professional phenomenon that is set by the World Health Organization (8).

Lebanon has experienced one of the most disastrous times with breakdown of various sectors such as economic, financial, health, and social (9). The precipitous decline of the national currency by over 80%, the 200% increase in prices, the deterioration of the financial industry, and the exodus of competent workers are all problems that the country faces (9). Additional difficulties include shortages of medicine, rising drug prices, high cost of treating chronic diseases, and a sharp spike in prices of all oil derivatives. The Beirut port explosion in August 2020 claimed the lives of over 220 people and destroyed the city. The nation had previously been impacted by numerous civil wars, an influx of Palestinian and Syrian refugees, establishments of camps for displaced people, and an increase in unemployment and poverty (9). All these elements trigger burnout and distress among postgraduate medical trainees (PGMT) (comprising residents and fellows).

Wellbeing is an imperative occupational strength since it is linked to employee satisfaction and effectiveness, safety behavior (10), proper functional management and organizational success. Thus, it is crucial to pay attention to the wellbeing of PGMT especially in a country that has all those challenges to maintain resource-efficient training and education (11). By such, it is important that leaders do not overlook the wellbeing of trainees. The primary aim of the study is to assess the sociodemographic features of PGMT and their connection to burnout by a validated and well-recognized questionnaire called the Oldenburg Burnout Inventory (OLBI) and by adding questions related to burnout and wellness activities. The secondary objective is to generate wellness initiatives for leaders to follow to combat burnout. The following study aims to expose the several unresolved issues in burnout research in healthcare among PGMT especially in an unstable country and how this can be compared to other countries and how the results can help prevent burnout and promote wellbeing.

## Methods

The study was conducted in a tertiary medical facility with 20 residency training programs in Beirut, Lebanon. The teaching team includes a program director, a program coordinator, and staff from the Graduate Medical Education office. For some programs, the years of training range from Post Graduate Year (PGY) 1 through PGY5 (the digits reflect the year of training, for example, PGY1 implies first year); for surgical sub-specialties, the years of training range from PGY1 to PGY6. The seventh year (PGY7) is for fellows. The programs are accredited by the Accreditation Council for Graduate Medical Education – International (ACGME-I). The medical center has been negatively impacted by the outbreak, collapse of the economy, port explosion in addition to other conditions. All parts of the structured training program have been unfavorably affected due to the interruption of cross-hospital rotations and missed education agendas, termination of elective surgery, decreased outpatient clinics and relocation of PGMT to other obligations during the pandemic.

The Institutional Review Board (IRB) of the university approved the study. The Lime Survey portal was utilized to send the emails. This portal was provided by the institution after securing consent from the IRB to conduct the study. The names and emails of PGMT were provided by the Graduate Medical Education office that possesses the files of all PGMT in the institution. The names and emails were not shared to the research team but only to the employee responsible for the Lime Survey. Three additional reminders were sent 1 week after the initial email. The inclusion criteria were met by male and female PGMT aged 18 years old or above, hired by the medical center, and who gave their consent to take part in the survey. The email included the names of the research team with the objectives of the study. It mentioned that the survey was voluntary and anonymous, and that it did not contain any personal identifiers. Those who were interested in participating were instructed to click the link in the email. There was no written consent to prevent gathering participant identifying information. Prior to finishing the survey, the participants had to confirm their agreement to participate. Valid surveys were integrated in the analysis and missing surveys were omitted.

The questionnaire was anonymous and web-based to calculate burnout by the OLBI (12). This instrument is available for non-commercial research use. The OLBI is a validated tool and includes two dimensions: disengagement and exhaustion. The two dimensions have 16 questions in total while each one has eight questions. There are four items which are phrased negatively, and four items which are phrased positively in each dimension. Ratings are done based on a Likert Scale from one to four (1 = strongly agree, 2 = agree, 3 = disagree, 4 = strongly disagree) (12). Items marked with “R” are reversed before the average scores for each sub-scale are measured in this way (1 = 4, 2 = 3, 3 = 2, 4 = 1). The cut-off scores for the OLBI are exhaustion ≥2.85 and disengagement ≥2.6 for very high burnout of personnel set by the author of the inventory (10). Moreover, we added questions after the OLBI to investigate some parts that were not captured by the OLBI. The participants had to answer these questions after completing the main survey and could not submit prior to completing these questions. The first question investigated whether trainees would report if they had burnout and if they answered yes, they were asked who they would report to. The next question was to choose three reasons for burnout from mentioned options that were given for them to select, and the next question was to indicate useful ways to lessen burnout from mentioned options that were given for them to select, and the last part was to list wellness programs that they considered helpful and beneficial during uncertain times. This question was open-ended wherein the participants could write and express their insights.

To ensure accuracy, the data was gathered in Microsoft Excel using the double-entry method. The Statistical Package for the Social Sciences (SPSS) (IBM Corp. Released 2020. IBM SPSS Statistics for Windows, Version 28.0. Armonk, NY: IBM Corp) was utilized for statistical analysis. To assess the internal consistency of the survey items, Cronbach’s alpha was used. The dependent variable is burnout presented with both dimensions (disengagement and exhaustion). The independent variables are gender, relationship status and year of training. Since the sample age range (26–32) was narrow, it was not included in our analysis because a preceding study done in Lebanon did not find any association between age and burnout (13).

In the descriptive analysis, frequencies and percentages were utilized for categorical data and mean and standard deviation (SD) for continuous data. We used the one-way analysis of variance (ANOVA) to compare between three groups or more, and the independent t-test to compare means between two groups. To determine the strength of the correlation between the demographic data and the dimensions, binary logistic regression was used. A *p* value of <0.05 is considered statistically significant. The overall Cronbach’s alpha for the inventory is 0.754.

### Patient and public involvement

There was no patient or public involvement in any way in this study.

## Results

After removing missing items, which resulted in the exclusion of every variable with a single missing value, the final valid number contained 188 participants (response rate = 48.32%). The descriptive data are shown in Table 1. PGMT comprise male and female residents or fellows working in the institution at the time of the survey and are above 18 years old. The sample is constituted by females 113 (60.1%) and males 75 (39.9%). Almost half of the sample is single (55.3%), 26.6% in a relationship, 17.0% married and 1.1% divorced. Table 2 indicates the complete scales seen in frequencies, percentages, mean and SD scores. The three questions with the highest mean are: “There are days when I feel tired before I arrive at work” (mean = 3.39), “After my work, I usually feel worn out and weary” (mean = 2.95) and “I can tolerate the pressure of my work very well” (mean = 2.91).

**Table 1:** Demographic characteristics of the trainees (*N* = 188).

	Frequency (%)
Gender	
Females	113 (60.1)
Males	75 (39.9)
Relationship status	
Single	104 (55.3)
In a relationship	50 (26.6)
Married	32 (17.0)
Divorced/separated	2 (1.1)
Year of training	
PGY1	52 (27.7)
PGY2	26 (13.8)
PGY3	42 (22.3)
PGY4	35 (18.6)
PGY5	17 (9.0)
PGY6	14 (7.4)
PGY7	2 (1.1)

**Table 2:** Frequency distribution, mean and SD of the OLBI (*N* = 188) (® = reversed question).

		Frequency (%)				Mean	SD
		Strongly agree	Agree	Disagree	Strongly disagree		
1	I always find new and interesting aspects in my work	32 (17.0)	121 (64.4)	33 (17.6)	2 (1.1)	2.03	0.62
2	There are days when I feel tired before I arrive at work®	86 (45.7)	91 (48.4)	10 (5.3)	1 (0.5)	3.39	0.61
3	It happens more and more often that I talk about my work in a negative way®	45 (23.9)	74 (39.4)	57 (30.3)	12 (6.4)	2.19	0.87
4	After work, I tend to need more time than in the past in order to relax and feel better®	86 (45.7)	62 (33.0)	40 (21.3)	0 (0.0)	1.76	0.78
5	I can tolerate the pressure of my work very well	6 (3.2)	36 (19.1)	114 (60.6)	32 (17.0)	2.91	0.69
6	Lately, I tend to think less at work and do my job almost mechanically®	25 (13.3)	74 (39.4)	75 (39.9)	14 (7.4)	2.59	0.81
7	I find my work to be a positive challenge	24 (12.8)	125 (66.5)	38 (20.2)	1 (0.5)	2.09	0.58
8	During my work, I often feel emotionally drained®	37 (19.7)	83 (44.1)	64 (34.0)	4 (2.1)	2.81	0.76
9	Over time, one can become disconnected from this type of work®	25 (13.3)	84 (44.7)	73 (38.8)	6 (3.2)	2.68	0.74
10	After working, I have enough energy for my leisure activities	7 (3.7)	57 (30.3)	74 (39.4)	50 (26.6)	2.89	0.84
11	Sometimes I feel sickened by my work tasks®	31 (16.5)	103 (54.8)	49 (26.1)	5 (2.7)	2.85	0.71
12	After my work, I usually feel worn out and weary®	40 (21.3)	99 (52.7)	49 (26.1)	0 (0.0)	2.95	0.68
13	This is the only type of work that I can imagine myself doing	62 (33.0)	66 (35.1)	50 (26.6)	10 (5.3)	2.04	0.90
14	Usually, I can manage the amount of my work well	42 (22.3)	129 (68.6)	17 (9.0)	0 (0.0)	1.87	0.54
15	I feel more and more engaged in my work	20 (10.6)	111 (59.0)	52 (27.7)	5 (2.7)	2.22	0.66
16	When I work, I usually feel energized	15 (8.0)	110 (58.5)	58 (30.9)	5 (2.7)	2.28	0.64

The prevalence rates of high burnout are 37.2% by disengagement and 51.1% by exhaustion. The independent t-test between the two dimensions and gender shows that there is no significant difference between the mean of disengagement and gender (*p* = 0.204). There is a significant difference between the mean of exhaustion and gender wherein the mean of female PGMT is 2.86 (SD = 0.35) versus the mean of male PGMT which is 2.69 (SD = 0.41) (*p* = 0.003).

The one-way ANOVA test between the mean of the two dimensions and relationship status shows that there is no significant difference between the mean of disengagement and relationship status (*p* = 0.477) and that there is no significant difference between the mean of exhaustion and relationship status (*p* = 0.105). On the other hand, Table 3 presents the one-way ANOVA test between the mean of the two dimensions and year of training and shows that there is a significant difference between the mean of disengagement and year of training with the mean of PGY3 being the highest (mean = 2.54; SD = 0.46), and the mean of PGY7 being the lowest (mean = 1.56; SD = 0.08), (*p* = 0.017). There is a significant difference between the mean of exhaustion and year of training with the mean of PGY3 being the highest (mean = 2.86; SD = 0.34) and the mean of PGY7 being the lowest (mean = 2.06; SD = 0.61), (*p* = 0.029). *Post hoc* analysis for the analysis of variance shows no significant differences between the different years of training when compared with the two dimensions.

**Table 3:** One-way ANOVA test between the mean of the two dimensions and year of training.

	Year of training	N	Mean	SD	*p* value
Disengagement	PGY1	52	2.35	0.48	0.017
	PGY2	26	2.48	0.56	
	PGY3	42	2.54	0.46	
	PGY4	35	2.47	0.43	
	PGY5	17	2.24	0.36	
	PGY6	14	2.25	0.38	
	PGY7	2	1.56	0.08	
Exhaustion	PGY1	52	2.85	0.33	0.029
	PGY2	26	2.82	0.47	
	PGY3	42	2.86	0.34	
	PGY4	35	2.75	0.40	
	PGY5	17	2.73	0.29	
	PGY6	14	2.60	0.44	
	PGY7	2	2.06	0.61	

Chi-square analysis is done between the cut-off points of the two dimensions and the different variables. In this case, 1 implies high burnout as per the cut-off points (exhaustion ≥2.85 and disengagement ≥2.6) (10). For the year of training, PGY6 and PGY7 are grouped together since PGY7 group includes only 2 participants and PGY6 group contains 14 participants. Their sum is used in this test to yield better results. According to the disengagement cut-off score, 41.6% of female trainees have high burnout and 30.7% of male trainees have high burnout (*p* = 0.166). According to the exhaustion cut-off score, 56.6% of female trainees and 42.7% of male trainees have high burnout (*p* = 0.074). According to the disengagement cut-off score, 34.6% of single trainees, 42.0% of those in a relationship and 40.6% of married trainees have high burnout (*p* = 0.544), while for the exhaustion cut-off score, 46.2% of single trainees, 62.0% of those in a relationship and 53.1% of married ones have high burnout (*p* = 0.136). According to the disengagement cut-off score, 30.8% of PGY1, 34.6% of PGY2, 52.4% of PGY3, 48.6% of PGY4, 23.5% of PGY5 and 12.5% of PGY6 and PGY7 trainees have high burnout (*p* = 0.027). The exhaustion cut-off score is not significant (*p* = 0.338).

Binary logistic regression is presented in Table 4. The references used are female for gender, single for relationship status and PGY1 for year of training. Compared to PGY1, the unadjusted odds ratio of burnout depicted by disengagement for PGY3 is 2.475 (*p* = 0.035). The remaining results are not statistically significant. After adjusting for relationship status, the odds ratio (0.621) for gender is kept the same for disengagement (p = 0.035). The same is noted when adjusting for year of training wherein the odds of ratio of gender is kept the same. After adjusting for relationship status, the odds ratio for gender (0.570) is kept the same for exhaustion (*p* = 0.062). The same is noted when adjusting for year of training wherein the odds of ratio of gender is kept the same.

**Table 4:** Unadjusted odds ratios for the two dimensions.

		Odd ratios	95% Confidence interval	*p* value
Disengagement	Males (Female = reference)	0.621	0.335–1.151	0.130
	In a relationship (Single = reference)	1.368	0.685–2.732	0.375
	Married	1.292	0.573–2.914	0.536
	Divorced	0.000	0.000	0.999
	PGY2 (PGY1 = reference)	1.191	0.438–3.237	0.732
	PGY3	2.475	1.063–5.760	0.035
	PGY4	2.125	0.876–5.157	0.096
	PGY5	0.692	0.195–2.455	0.569
	PGY6-7	0.321	0.065–1.583	0.163
Exhaustion	Males (Female = reference)	0.570	0.316–1.028	0.062
	In a relationship (Single = reference)	1.904	0.956–3.791	0.067
	Married	1.322	0.593–2.926	0.491
	Divorced	0.000	0.000	0.999
	PGY2 (PGY1 = reference)	1.167	0.454–2.997	0.749
	PGY3	1.471	0.647–3.345	0.358
	PGY4	1.059	0.449–2.495	0.896
	PGY5	1.125	0.376–3.368	0.833
	PGY6-7	0.333	0.095–1.170	0.086

Table 5 shows the additional questions that were addressed. Most residents are hesitant to report that they have burnout (65.4% versus 34.6%). For those who answered that they would report if they had burnout, most stated that they would inform their fellow residents or senior residents with a lesser extent the program director. The top reasons for burnout cited are working hours, salary, and working conditions. Reducing inefficient and administrative duties, receiving financial support, and having more vacations are stated as top reasons to help decrease burnout.

**Table 5:** Additional questions added after the OLBI.

Questions	Yes *N* (%)	No *N* (%)
If you ever feel burnout, will you report it? (*N* = 188)	65 (34.6%)	123 (65.4%)
	Multiple choice answers	%
If yes, then to who? (You can choose more than one) (*N* = 65)	Resident/fellow at the same level	25.5%
	Senior/chief resident/fellow	20.2%
	Program director	13.8%
	Program coordinator	5.3%
	Division head/chairperson	4.3%
Please list top three reasons for burnout from work (*N* = 188)	Working hours	72.9%
	Salary	57.4%
	Working conditions	50.0%
	Faculty members (not necessarily all)	41.0%
	Rotation structure	36.7%
	Other residents/fellows (not necessarily all)	35.1%
	Patients	22.9%
	Other staff who you regularly work with	21.8%
Which ways are useful to reduce the symptoms of burnout during uncertain times? (You can choose more than one) (*N* = 188)	Reducing inefficient work processes and non-physician clerical work	77.1%
	Receiving financial support (increase in salary/stipend) by the organization	73.4%
	Offering more annual leaves when needed	50.0%
	Reducing working hours (even less than 80 hours per week, averaged over a four-week period)	47.3%
	Implementing free wellness programs (yoga, exercises, swimming, hiking, etc.)	40.4%
	Receiving moral support by program director on regular basis during meetings	37.8%
	Social gatherings/outings in presence of program director and/or chairperson	32.4%
	Regularly assessing burnout through evaluations to be shared with program director	27.1%
	Regular online/physical lectures or discussions with mental health specialists	13.8%
	Providing personal protective equipment and training on safety measures during the COVID-19 pandemic	6.9%
	Answers	
Please list wellness programs that you consider helpful and beneficial during uncertain times (*N* = 106)	Most answers revolve around exercising, hiking and some wellness activities such as yoga or meditation or having some free time for oneself or for activities with friends. Other elements include childcare, activities involving music (dancing classes), and cognitive behavioral therapy. Additional items are support from seniors, group discussion, faculty meetings, financial support and better payment, flexible working hours/more breaks, and group activities (such as sightseeing, outings with the department team, etc.).	

## Discussion

This descriptive study aims to determine the prevalence of burnout and analyze its relationship with sociodemographic characteristics during the COVID-19 pandemic and the extreme conditions experienced by PGMT in Lebanon. The prevalence rate of high burnout among PGMT is 51.1% by exhaustion and 37.2% by disengagement. This is in lieu of the uncertain and challenging conditions in the country which have affected trainees dramatically. The questions from the OLBI with the highest mean scores revolve around feeling tired even before starting to work, feeling worn out after work even though they mentioned that they were able to tolerate the pressure of work.

Several other studies have utilized other inventories like the Copenhagen Burnout Inventory. A study of 113 PGMT in Pakistan showed that the mean of personal burnout was 49.74, while the mean of work-related burnout was 46.99, and the mean of client-related burnout was 46.13 (14). A different study conducted in India among 210 PGMT found that personal burnout was 51.8%, work-related burnout was 37.2% (calculated among 151 PGMT), and client-related burnout was 22.47% (calculated among 91 PGMT) (15). The prevalence of personal burnout was 41.6% in another study of 245 PGMT in Sri Lanka while the prevalence of work-related burnout was 30.6 and 8.9% for client-related burnout (16).

A study utilized the OLBI and showed that 116 psychiatric residents in Romania had the following burnout scores: high burnout in 22.4% of residents, moderate burnout in 51.7% of residents, and low burnout in 25.9% of residents, while 25% had high disengagement and 23.3% had high exhaustion (17). Different burnout scores suggest various explanations. The instruments used may partially account for the variation in the prevalence rate of burnout (18). Moreover, training specialty exhibits a drastic role in determining burnout. For instance, certain specialties have less emergency exposure or confrontations than other specialties, and hence may report less burnout (15).

The significant difference between the mean of exhaustion and gender indicates that female trainees are at higher risk of burnout or other mental health problems, as shown in many studies (19). Although 56.6% of female trainees report high burnout by exhaustion, the result is not statistically significant. The same is noted with disengagement wherein 41.6% of female trainees have high burnout, in comparison with males, but not statistically significant. This can be elucidated by disparities in gender characters, responsibilities and cultural variances (15) wherein female PGMT particularly married ones may report more burnout. This is due to exhaustion and overtiredness from their extra responsibilities and disruption of the work-life balance. The one-way ANOVA indicates a significant difference between both the mean of disengagement and the mean of exhaustion with year of training. Since 63.8% of trainees are in the first 3 years of training, this might partially explain the noted difference, meaning that trainees needed time to adjust and tolerate and get used to the training process.

The additional questions addressed revealed several concerns and limitations. Most trainees stated that they would not report if they had burnout, which might be due to lack of trust in leaders or lack of hope that any improvement will happen. Other trainees mentioned that there should be a proper reporting channel for unprofessional behavior instigated by peers or faculty members. This shows that leaders should work hard on attempting to gain the trust of trainees to ensure a smooth and ethical training process. Such an attitude may be partly due to the COVID-19 pandemic that brought up many issues in general and disrupted the normal behavior of professionals and progression of daily functions. Other challenges that trainees reported were low salaries, limited childcare for those who have family responsibilities, and decreased education and surgical training. This may be true in various medical institutions in the same country and perhaps in different countries. This had coincided with other published studies wherein 47% of participants were anxious about overlooked educational prospects (20). Another point to address was to minimize unnecessary administrative and clerical duties that are time-consuming and increase burnout among PGMT.

The following represents important quotes from the participants that are essential and can serve as a guideline for leaders to consider for empowerment of PGMT and for proper management of their education and training. This was the last additional question from the extra questions that we added after the OLBI and was open-ended wherein the participants had the freedom to express themselves and address any concern.

Fear of raising the issue of burnout due to concern from leaders, as one participant mentioned:

*“Reporting burnout to my attending in this institution has fired back negatively on me around 4 months ago and destroyed my evaluation. It is a shame. It even gave a picture of me being a “bad time manager.”*

Encouraging trainees to report unprofessional attitude in a confidential manner, as one participant mentioned:

*“Residents, particularly junior residents, and interns should be empowered to report unprofessional and downright toxic/malignant behavior from their senior residents and attendings. There needs to be an active effort in ridding certain departments of the toxic blame culture and stop normalizing hazing-like behavior.”*

Trainees do not perceive wellness activities as effective against mitigating burnout, as one participant reported:

*“Wellness programs are useless to be honest. They are meant to talk and talk with no action being done. I am stressed when I come to work. I do not have any stressors outside my work life. The salary does not help also nor do the attendings. Your chiefs think they can boss you around just because they are 1 year older. They do not listen, do not negotiate, do not help, and think we only live in the hospital. I have wanted to quit so many times because of the stresses of the workplace.”*

*“We can state infinite wellness programs, apply them, and then think that these have eradicated/reduced burnout and have created a better working environment. But the main objective of these feedback is to change and transform the way we educate our residents. Every resident would work his heart out if he is given, in return, the appropriate amount of medical experience and education. Hence the need for a proper patient load, which will create a better working environment.”*

Promoting good rapport with leadership:

*“Let us fix priorities before providing yoga classes. Good relationship with faculty members, constructive criticism, educational lectures. Reduction of clerkship work at the clinics and including clinical case discussion sessions. Engaging faculty members in teaching us rather than relying on the fellows to prepare all the lectures.”*

Increasing salary to make ends meet if funding is available:

*“Increasing salaries because we feel we are short of money and need to do extra work (which we cannot do), which makes us frustrated and demotivated. Second thing is getting the people to do activities aside from just working.”*

Another participant added:

*“Money helps. More money means I can have a nice meal, or travel or buy something I could not afford or have a nanny at home so I can rest. Money makes a difference.”*

Increasing patient load to enhance training:

*“More operations – working with patients/staff and doing unfulfilling tasks while having a diminishing OR exposure because of the financial situation is exhausting.”*

Providing childcare for trainees with children and family responsibilities:

“On-site day care for our kids when we take 10 min off to do pumping for our newborns at home it is okay if we work less than 80 h a week; does not make us bad doctors. All the surveys and the talks are useless; do something about it.”

### Practical implications

We have outlined potential support strategies to preserve trainees’ wellbeing based on the above results.

- Educational support: well-organized and disciplined rotations among specialties and sponsoring institutions; wide-ranging instructive curriculum; advanced skills training in the operating room and medical wards; committed laboratory or wet/dry skills lab training sessions.
- Training support: reliable coverage schedules; flexible hours for those who have burnout symptoms and financial support for additional training, research, and academic conferences.
- Social support: continuous appreciation to elevate morale and productivity; support for partners and dependents; accessible childcare assistance; meal support and discounts on available programs (gym membership, food services, home appliances, etc).
- Moral support: personalized contact with leaders (open door policy, attentive listening, and problem-solving skills), and available counseling services. Open communication and awareness about mental health, wellbeing, and burnout.

This could be achieved by attempting to reduce hospital fees to acceptable costs to increase the patient load and promote the possibility for learning and practicing. Leaders should focus on positive reinforcement by (1) eliminating the old school non-constructive negative feedback, (2) giving mandatory lectures to faculty members on how to deal with trainees’ concerns and engaging in proper problem management and positive communication, and (3) investing in surgical apprentice on cadavers, cadaveric dissection, and simulation labs for any kind of interventions. This would set more frequent faculty member to resident “give and take” relationships, wherein both ends would fulfill their roles to one another with mutual respect. In other words, PGMT should feel supported by their institution, and the latter should invest in enhancing their medical experience, confidence, and training, to finally graduate excellent doctors.

Strategies could include de-stigmatizing mental health and normalizing its access. At this point, creating health initiatives such as supportive workplace environment with coworkers does not seem appealing or welcoming, and thus leaders should resort to other options. Our study has shown that PGMT opt for practical solutions and not lectured based interventions. They want pragmatic solutions that will specifically benefit their educational experience and in turn ameliorate their wellbeing. The above quotes illustrate how their top priorities are education and experience and not mindfulness activities. This requires dedicated, structured, and constructive reforming of the wellness curriculum. Leaders should work extensively in trying to adopt practices that aim to involve faculty members in teaching process and apply assessment wherein PGMT evaluate faculty in a confidential way while attempting to address pitfalls. This can also entail regular meetings with PGMT and faculty members to increase mentoring and yield a better teaching experience. By encouraging good interpersonal interactions, prompt problem solving, and operational planning, leaders could increase trainee operational autonomy and empowerment.

Future research should examine how wellness initiatives affect burnout before, during, and after the COVID-19 outbreak, compare burnout across specialties and highlight the influence of wellness proposals and their consequences on wellbeing. Attention must be given to the following areas: identifying burnout, giving PGMT the confidence to approach their leaders for support, establishing workload, enhancing scheduling, and working environment, and supporting psychological and mental wellbeing. More research is essential to address many unresolved concerns, such as uncovering all potential strategies that trainees may use to prevent burnout or mitigate its effects (21).

The study has several limitations. The Maslach Burnout Inventory (1) or the Copenhagen Burnout Inventory (14) have been used in research to measure burnout and this could yield different results. The study was performed in one medical center, and this limits generalization to other centers that have similar training programs. We can only identify correlations because the cross-sectional approach does not assess burnout over time. The lack of regular comparisons of outcomes from pandemics such as before, during and after the outbreak could be useful but difficult to get. Selection bias, or the possibility that survey completion was more likely done by trainees with free time, can result in underreporting of burnout. Response bias occurs when participants with burnout are less prone to completing the survey. Participants may respond in a way that they believe others would prefer them to, which causes reporter bias. Due to the difficult circumstances, PGMT can be hesitant or discouraged from completing surveys, thus, we cannot determine whether the participants provided honest responses, but we can only assume so.

## Conclusion

Burnout among PGMT is a critical concern, especially in a country that has enfolded in a crisis and is struggling to survive among many other challenges. The COVID-19 outbreak has interrupted all sections of day-to-day life, especially the healthcare sector which is at the front of this turmoil. There is a necessity to mitigate burnout among PGMT during such difficult times. It is the duty of leaders to recognize and foster a culture and environment where wellbeing can prosper to support PGMT at this stage. These measures comprise offering psychological and moral support, setting better working hours, adjusting salaries, and providing better teaching and training options.

## Data availability statement

The raw data supporting the conclusions of this article will be made available by the authors, without undue reservation.

## Ethics statement

The studies involving human participants were reviewed and approved by the Institutional Review Board (IRB) of the American University of Beirut Medical Center (AUBMC) (SBS-2021-0219). The ethics committee waived the requirement of written informed consent for participation.

## Author contributions

AE, JD, and AH: conceptualization, methodology, formal analysis, investigation, and writing—original draft preparation. AY, JD, ED, and AE: validation, project administration, visualization, and writing—review and editing. ED and AE: resources and supervision. AY and ED: data curation. All authors contributed to the article and approved the submitted version.

## Conflict of interest

The authors declare that the research was conducted in the absence of any commercial or financial relationships that could be construed as a potential conflict of interest.

## Publisher’s note

All claims expressed in this article are solely those of the authors and do not necessarily represent those of their affiliated organizations, or those of the publisher, the editors and the reviewers. Any product that may be evaluated in this article, or claim that may be made by its manufacturer, is not guaranteed or endorsed by the publisher.
